# A Novel Bioinformatics Method for Efficient Knowledge Discovery by BLSOM from Big Genomic Sequence Data

**DOI:** 10.1155/2014/765648

**Published:** 2014-04-03

**Authors:** Yu Bai, Yuki Iwasaki, Shigehiko Kanaya, Yue Zhao, Toshimichi Ikemura

**Affiliations:** ^1^Graduate School of Information Science, Nara Institute of Science and Technology, 8916-5 Takayama-cho, Ikoma-shi, Nara 630-0192, Japan; ^2^Department of Bioscience, Nagahama Institute of Bio-Science and Technology, Nagahama-shi, Shiga-ken 526-0829, Japan; ^3^Sino-Dutch Biomedical and Information Engineering School, Northeastern University, Shenyang, Liaoning 110004, China

## Abstract

With remarkable increase of genomic sequence data of a wide range of species, novel tools are needed for comprehensive analyses of the big sequence data. Self-Organizing Map (SOM) is an effective tool for clustering and visualizing high-dimensional data such as oligonucleotide composition on one map. By modifying the conventional SOM, we have previously developed Batch-Learning SOM (BLSOM), which allows classification of sequence fragments according to species, solely depending on the oligonucleotide composition. In the present study, we introduce the oligonucleotide BLSOM used for characterization of vertebrate genome sequences. We first analyzed pentanucleotide compositions in 100 kb sequences derived from a wide range of vertebrate genomes and then the compositions in the human and mouse genomes in order to investigate an efficient method for detecting differences between the closely related genomes. BLSOM can recognize the species-specific key combination of oligonucleotide frequencies in each genome, which is called a “genome signature,” and the specific regions specifically enriched in transcription-factor-binding sequences. Because the classification and visualization power is very high, BLSOM is an efficient powerful tool for extracting a wide range of information from massive amounts of genomic sequences (i.e., big sequence data).

## 1. Introduction

Genome sequences, both protein coding and non-coding parts of the sequences, contain a wealth of information. The G + C content (G + C%) is a fundamental characteristic of individual genomes and used for a long period as a basic phylogenetic parameter to characterize inter- and intragenomic differences. The G + C%, however, is too simple to differentiate wide varieties of genomes. Many groups have reported that the oligonucleotide composition, which is an example of high-dimensional data, varies significantly among genomes and can be used to study genome diversity [1–9], and the oligonucleotide compositions, including dinucleotide composition, are called the “genome signature” of each species. Various linguistic tools for analyzing DNA sequence have been developed [8, 9]. Unsupervised neural network algorithm, Kohonen's Self-Organizing Map (SOM), is a powerful tool for clustering and visualizing high-dimensional complex data on a two-dimensional map [10–12]. On the basis of batch learning SOM, we have previously developed a modification of the conventional SOM for genome and gene sequence analyses, which makes the learning process and resulting map independent of the order of data input: BLSOM [13–15]. Importantly, BLSOM is suitable for actualizing high-performance parallel-computing and, therefore, can analyze big sequence data such as millions of genomic sequences simultaneously [16].

When we constructed BLSOMs for di-, tri-, and tetranucleotide composition in 10 kb genomic sequences derived from a wide range of prokaryotic and eukaryotic genomes, the sequences were clustered (i.e., self-organized) according to species without any information regarding the species during the BLSOM calculation, and increasing the length of the oligonucleotides from di- to tetranucleotides increased the clustering power [15]. An apparent causative factor for the genome signature is the context-dependent DNA mutation and repair mechanisms. It should also be noted that oligonucleotides especially longer than trinucleotides often represent motif sequences responsible for sequence-specific protein binding (e.g., transcription factor binding). The occurrence of such motif oligonucleotides in the genome should differ from the level expected from the mononucleotide composition in the respective genome and may differ among genomic portions of one genome. We have recently found that DegPenta and DegHexa for the human genome can effectively detect characteristic occurrence patterns of many transcription-factor-binding motifs in pericentromeric heterochromatin regions [17].

In the present study, in order to clarify vertebrates' genome signatures, we first analyzed pentanucleotide compositions in 100 kb genomic sequences derived from a wide range of vertebrates and then those from human and mouse genomes in order to investigate the power to detect differences between the closely related genomes.

## 2. Materials and Methods

### 2.1. BLSOM

BLSOM is an unsupervised neural network algorithm that implements a characteristic nonlinear projection from the high-dimensional space of input data onto a two-dimensional array of weight vectors [10, 12]. We have previously modified the conventional SOM for genome informatics to make the learning process and resulting map independent of the order of data input and established BLSOM [13–15]. Here, we explain the BLSOM method developed by Kanaya et al. [13].

In the original Kohonen's SOM, the initial vectorial data were set by random values, but in the BLSOM the initial vectors are set based on the widest scale of the sequence distribution in the oligonucleotide frequency space with the principal component analysis (PCA) [13]. Weights in the first dimension (*I*) were arranged into lattices corresponding to a width of five times the standard deviation (5*σ*_1_) of the first principal component: the second dimension (*J*) was defined by the nearest integer greater than *σ*_2_/*σ*_1_ × *I*; and *I* was set in the present study as the average number of sequence data per neuron which becomes approximately four. *σ*_1_ and *σ*_2_ were the standard deviations of the first and second principal components, respectively. The weight vector on the *ij*th lattice (**w**_*ij*_) was represented as follows:

(1) $$\begin{array}{c} w_{ij}=x_{\text{av}}+\frac{5\sigma_{1}}{I}\left[b_{1}\left(i-\frac{I}{2}\right)+b_{2}\left(j-\frac{J}{2}\right)\right], \end{array}$$

where **x**_av_ is the average vector for oligonucleotide frequencies of all input vectors, and **b**_1_ and **b**_2_ are eigenvectors for the first and second principal components. In Step 2, the Euclidean distances between the input vector **x**_*k*_ and all weight vectors **w**_*ij*_ were calculated; then **x**_*k*_ was associated with the weight vector (called **w**_*i*′*j*′_) with minimal distance. After associating all input vectors with weight vectors, updating was done according to Kanaya et al. [13].

BLSOM learning for oligonucleotide composition was conducted as described previously [15]. BLSOM program was obtained from Niigata Univ. (takaabe@ie.niigata-u.ac.jp) or UNTROD, Inc. (y_wada@nagahama-i-bio.ac.jp).

### 2.2. *U*-Matrix

Distances of weight vectors between neighboring lattice points on BLSOM can be visualized as black levels with a *U*-matrix method [19], and this provides information regarding similarity of oligonucleotide composition in local areas on BLSOM; the areas composed of lattice points with similar or distinct oligonucleotide composition can be recognized as low or high black level, respectively.

### 2.3. Genome Sequences

Genome DNA sequences were obtained from UCSC ftp site (http://www.ncbi.nlm.nih.gov/genomes/). When the number of undetermined nucleotides (Ns) in a fragment sequence (e.g., 100 kb) exceeded 20% of the sequence, the sequence was omitted from the analysis. In the case where the number of Ns was less than 20%, the oligonucleotide frequencies were normalized to the length without Ns and included in the analysis.

## 3. Results

### 3.1. Characteristics of BLSOM Clustering

In the era of extensive genome sequencing, it is important to develop novel bioinformatics tools to support an efficient knowledge discovery from massive amounts of genomic sequences. Analyses on the species-specific oligonucleotide composition “genome signature” (e.g., penta- and hexanucleotide compositions) may provide* in silico* information concerning important signal sequences such as transcription factor binding sequences [17]. To show the clustering ability of BLSOM for vertebrate genome sequences and to explain the basal features of BLSOM clustering patterns, we first analyze pentanucleotide compositions in 100 kb sequence fragments derived from 10 vertebrate genomes.

In DNA databases, only one strand of each pair of complementary sequences is registered. Previous analysis of prokaryotic species that was done by Abe et al. [15] revealed that sequences (e.g., 10 kb sequences) from a single prokaryotic genome were often split vertically into two territories according to the transcriptional direction of the genes present in the fragment. However, to study general characteristics of genomic sequences such as the genome signature, differences in the oligonucleotide composition between two complementary strands are not necessarily important. Therefore, we construct a BLSOM in which the frequencies of a pair of complementary pentanucleotides (e.g., AAAAC and GTTTT) in each fragment are summed [17]. The BLSOM for this degenerate set of a pair of complementary pentanucleotides is designated as DegPenta.

**Figure 1:**
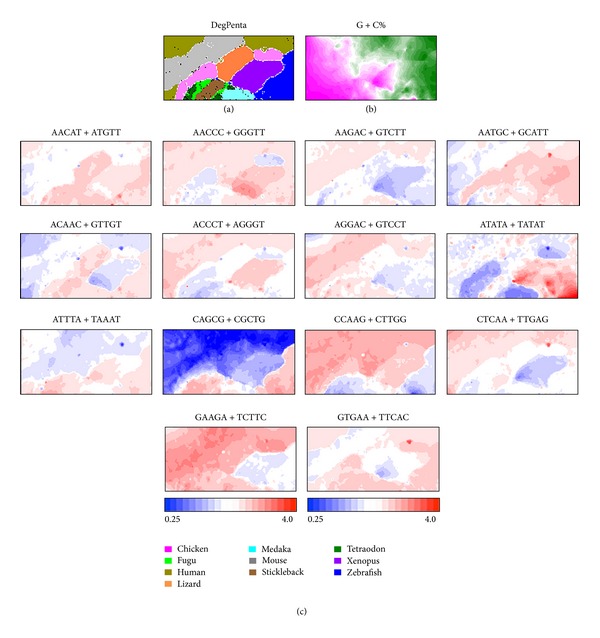
BLSOMs for 100 kb sequences derived from 10 vertebrata genomes. (a) DegPenta. Lattice points containing sequences from multiple species are indicated in black and those containing sequences from a single species are indicated in color as shown in the keys. (b) G + C%. For each lattice point in the DegPenta, G + C% was calculated and divided into 21 categories with an equal number of lattices. The lattice points belonging to the categories of the highest, middle, and lowest G + C% are shown in wine red, white, and green, respectively. (c) Diagnostic pentanucleotides responsible for species-specific clustering. Occurrence of each pentanucleotide for each lattice point was calculated and normalized with occurrence expected from the mononucleotide composition for the respective lattice point [16, 17]. This observed/expected ratio is indicated in color presented under the panel. This ratio has been shown to be useful in unveiling genome signatures because the oligonucleotide composition can be analyzed independently of a simplex effect reflecting the mononucleotide composition of genomic sequences [16–18].

On the BLSOM, lattice points containing sequences from a single species are indicated in a color specifying the species; those containing sequences from multiple species are indicated in black. Because most lattice points are colored, a high separation power is apparent for DegPenta (Figure 1(a)), with no information concerning species during the BLSOM calculation. We next explain the basal characteristics of BLSOM separation observed for the vertebrate sequences. G + C% has long been used as a fundamental value that characterizes both inter- and intragenomic differences. For example, on a warm-blooded vertebrate genome, there exists a long-range segmental G + C% distribution “isochores,” which have been connected with chromosomal bands [7, 20–23]. Figure 1(b) presents the G + C% that is calculated from pentanucleotide composition at each lattice point in the DegPenta. Sequences with high and low G + C% (wine red or green in Figure 1(b)) are located on the left and right side of the map, respectively, showing that the G + C% level is reflected primarily in the horizontal direction. The territory of each species is often split into several subterritories, which should relate at least in part to isochore structures because the G + C% level differs between subterritories of a single species, for example, chicken and human territories.

BLSOMs can visualize diagnostic oligonucleotides responsible for species-specific clustering (self-organization). We first calculate the pentanucleotide frequencies expected from the mononucleotide composition that is obtained from the vectorial data (i.e., pentanucleotide composition) at each lattice point and indicate the observed/expected ratio as follows: red (overrepresented), blue (underrepresented), and white (moderately represented) (Figure 1(c)). This observed/expected ratio is useful in unveiling genome signatures, since it allows us to examine the oligonucleotide composition at each lattice point, independently of a simple effect derived from its mononucleotide composition [17]. For various pentanucleotides, transitions between red and blue often coincide exactly with species-specific territory borders. AACAT + ATGTT, ACAAC + GTTGT, ATTTA + TAAAT, and CAGCG + CGCTG are overrepresented in fishes (Fugu, Medaka, Stickleback, Tetraodon, and Zebrafish) but not in almost all tetrapods (Human, Lizard, Mouse, Chicken, and Xenopus). ACCCT + AGGGT and CCAAG + CTTGG are overrepresented in tetrapods but not in fishes. AACCC + GGGTT is underrepresented in chicken and a part of fish (Fugu, Stickleback, and Tetraodon). GAAGA + TCTTC is underrepresented in Xenopus and Zebrafish. These findings show that BLSOM can recognize the species-specific oligonucleotide composition and identify the combinatorial diagnostic oligonucleotides responsible for species-specific clustering; that is, a combination of not a few but many pentanucleotides contributes to the accurate clustering (self-organization) of genomic sequences according to species.

### 3.2. BLSOMs for Human and Mouse Genomes

**Figure 2:**
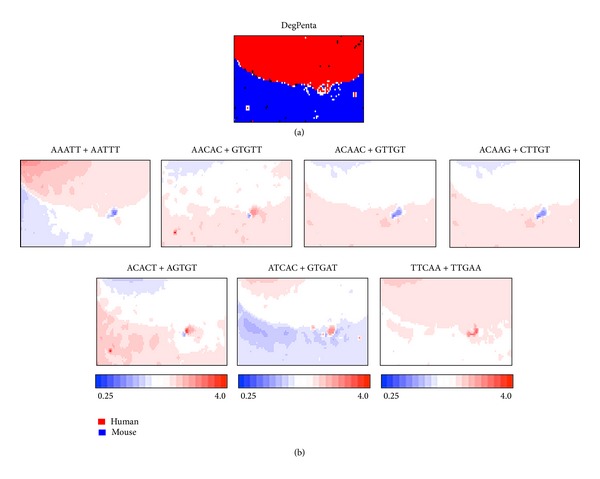
BLSOMs for 100 kb sequences derived from the human and mouse genomes. (a) DegPenta. Lattice points containing sequences from human and mouse are indicated in black and those containing sequences from a single species are indicated in color as shown in the keys. (b) Occurrence level of each pair of complimentary pentanucleotides in the DegPenta. Level of a complimentary pentanucleotide pair for each lattice point is calculated and normalized with the level expected from the mononucleotide composition for the lattice point. The observed/expected ratio is indicated in colors presented at the bottom of the figure. Seven examples of the pentanucleotides diagnostic for species-specific separations are presented.

We have next constructed DegPenta with 100 kb sequences derived from the human and mouse genomes (Figure 2). This enables us to examine a BLSOM power for separating the species with a relatively close phylogenetic relationship and to clarify difference in the genome signatures of the closely related species. Lattice points that contain sequences derived from human and mouse are indicated in red and blue, respectively, and those that include sequences from both human and mouse are indicated in black. With no information regarding species during the BLSOM calculation, the species-specific clustering (self-organization) of the 100 kb sequences is clear.

In Figure 2(b), the observed/expected ratios of individual pentanucleotides calculated as explained in Figure 1(c) are illustrated in red (overrepresented), blue (underrepresented), and white (moderately represented). Transitions between red (overrepresentation) and blue (underrepresentation) for various pentanucleotides often coincide exactly with species territory borders, showing that BLSOM recognizes the species-specific combination of oligonucleotide frequencies that is the representative signature of one genome and enables us to identify the frequency patterns that are characteristics of individual genomes.

Seven examples of the pentanucleotides diagnostic for the species territory formation are presented (Figure 2(b)). AAATT + AATTT, ATCAC + GTGAT, and TTCAA + TTGAA are preferred in the human genome but not in the mouse genome. On the other hand, AACAC + GTGTT, ACAAC + GTTGT, ACAAG + CTTGT, and ACACT + AGTGT are preferred in the mouse genome but not in the human genome. It should be stressed that a complex combination of many pentanucleotides contributes to the species-specific clustering (i.e., self-organization) of sequence fragments. Some of these diagnostic pentanucleotides, if not all, may have important biological significances, which should be related to functions.

### 3.3. Characteristics of Sequences Belonging to Specific Zones

While most 100 kb sequences are classified primarily into species-specific territories, there are a few minor human zones (red) that are located within the mouse territory (blue) and are surrounded with white lattice points. In addition, there is a nub-type human zone that is located in the border region between human and mouse territories and also is surrounded by white lattice points. In Figure 2(a), lattice points with no genomic sequence assigned after the BLSOM calculation are left white. It should be mentioned that Abe et al. [15] and Iwasaki et al. [17] have previously shown that lattice points containing genomic sequences whose oligonucleotide composition is very distinct from other genomic sequences tend to be surrounded by lattice points containing no genomic sequence.

**Figure 3:**
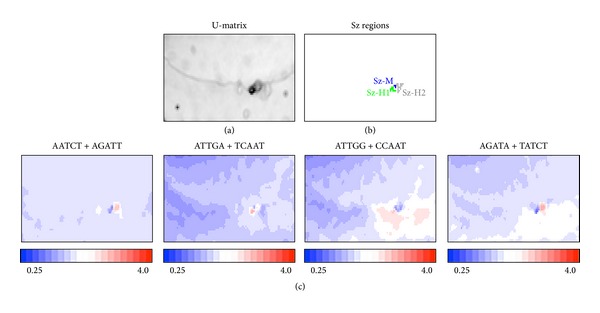
Pentanucleotides specifically enriched in specific region. (a) *U*-matrix for the BLSOM listed in Figure 2(a). (b) Sz regions. Sz-H1 and Sz-H2 regions of human sequences are indicated in green and gray letters, respectively. A very tiny Sz-M region of mouse sequences is indicated in blue. (c) Pentanucleotides specifically enriched in Sz. The observed/expected ratio for each pair of complimentary pentanucleotides is calculated as described in Figure 1(c) and indicated in colors presented under the panel.

Similarity in oligonucleotide composition between neighboring lattice points in BLSOM (and thus between sequences belonging to neighboring lattice points) can be visualized using a *U*-matrix [19] with a level of blackness (Figure 3(a)), as described in Section 2. On the *U*-matrix, borders between human and mouse territories are visualized as black lines, which represent distinct pentanucleotide compositions between human and mouse sequences. Furthermore, there are small dark black zones and gray zones surrounded by a black circle, which should contain sequences with peculiar oligonucleotide composition distinct from the compositions from other genomic sequences; the respective zones composed of human sequences are numbered as Sz-H1 and Sz-H2 and that of mouse sequences is specified as Sz-M (Figure 3(b)). Importantly, these numbered zones primarily correspond to zones surrounded by white lattice points in Figure 2(a), confirming that the sequences in these specific zones have peculiar oligonucleotide compositions very distinct from a major portion of the respective genome. Actually, occurrence levels of individual pentanucleotides in the specific zones are clearly different from those in the major portion of the respective genome (Figure 2(b)). AATCT + AGATT and AGATA + TATCT are preferred in Sz-H2 but not in Sz-H1 and Sz-M. ATTGA + TCAAT is preferred in Sz-H1 and Sz-H2 but not in Sz-M. ATTGG + CCAAT is preferred in Sz-H1 but not in Sz-H2 and Sz-M. The pentanucleotides listed in Figure 2(b) correspond to human transcription-factor-binding (TFB) motifs and the reason why these motif pentanucleotides are chosen is explained below.

The oligonucleotides such as penta- and hexanucleotides often provide the binding sites of proteins such as transcription factors. When we consider the oligonucleotides that can function as important signal sequences such as TFB motifs, their occurrence levels in genomic sequences should be biased significantly from the levels expected from random sequences. Therefore, the overrepresentation of a certain oligonucleotide only in a restricted portion of the BLSOM (and thus of the genomic sequences) is thought to provide useful information for understating the biological significance of the respective sequence, especially when a biological function of the oligonucleotide of interest is known.

In our previous study [17], we have shown that oligonucleotide BLSOM such as DegPenta can be used for studying sequences derived even from one genome. In that study, addition of computer-generated random sequences to real human sequences can successfully separate the specific sequences with distinct oligonucleotide composition from a major portion of the human genome; that is, these specific sequences are displaced well from the major portion of human sequences and surrounded by the random sequences. Interestingly, the specific human sequences thus found are derived mainly from pericentromeric regions and enriched by TFB motif sequences [17]. Instead of the human plus random sequences used in the previous study, human plus mouse sequences are analyzed in the present study, and the addition of the closely related species appears to effectively assign the 100 kb sequences with peculiar oligonucleotide compositions very distinct from those in the major portion of the respective genome (Figure 2(a)). In order to clarify the characteristics of the specific sequences found in this study and to compare with those found previously, we have analyzed the occurrence of the pentanucleotides corresponding to human TFB motifs analyzed in the previous paper. All of the TFB pentanucleotides are overrepresented (red) in a certain specific zone but underrepresented (blue) in almost all other human 100 kb sequences, confirming the previous result. When we examine their occurrences in the mouse territory, AATCT + AGATT, ATTGA + TCAAT, and TATCA + TGATA are underrepresented in a major portion of the mouse genome. However, ATTGG + CCAAT and AGATA + TATCT are underrepresented only in a half portion of the mouse genome, indicating that the biological function of these two pentanucleotides may differ from that for human. Comparative analyses of the closely related species can provide this type of information concerning a possible evolutionary change in functional signal sequences such as TFB motif sequences, but the addition of the computer-generated ransom sequences cannot provide the information concerning the molecular evolutionary change. The reason why the specific zones of mouse on DegPenta are less evident than human will be discussed below.

## 4. Discussion

### 4.1. Repeat and Unique Sequences

Vertebrate genomes are composed of repeat and nonrepeat, unique sequences, which have distinct biological functions. Since repeat sequences usually have peculiar oligonucleotide composition, there exists a possibility that the specific zones' sequences with peculiar oligonucleotide compositions distinct from a major portion of the genome are repeat sequences, and this possibility is examined as follows. In the UCSC database, repeat sequences identified by RepeatMasker and Tandem Repeats Finder are specified in lower-case letters for distinguishing from unique sequences specified in upper-case letters. We first concatenated unique or repeat sequences separately, divided these concatenated sequences into 100 kb sequences, and counted pentanucleotide composition in each 100 kb sequence.

**Figure 4:**
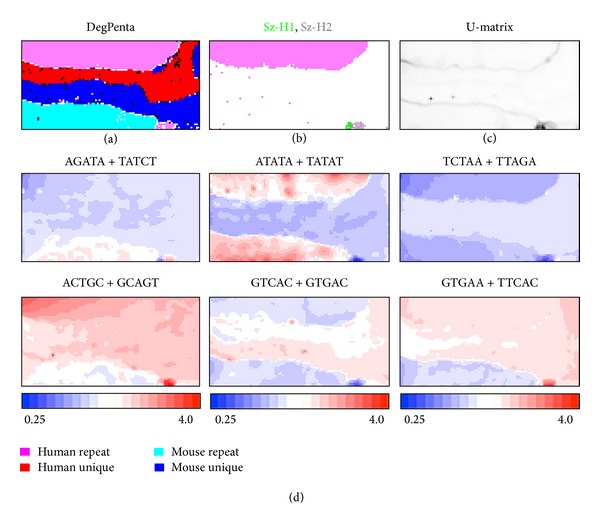
BLSOMs for 100 kb repeat and unique sequences derived from the human and mouse genomes. (a) DegPenta. Lattice points containing sequences from more than one category are indicated in black and those containing sequences from a single category are indicated in color as shown in the keys. (b) Human repeat sequences. Human Sz-H1 and Sz-H2 sequences defined in Figure 3(b) are mapped and indicated in green and gray, respectively. (c) *U*-matrix for the DegPenta listed in (a). (d) Diagnostic pentanucleotides responsible for species-specific clustering. The observed/expected ratio for each pair of complimentary pentanucleotides is calculated as described in Figure 1(c) and indicated in color presented under the panel.

Clear separation between species and between repeat and unique sequences is observed on DegPenta (Figure 4(a)). Interestingly, human repeat sequences (pink) forms one satellite-type minor territory located at the lowest part of the map and the mapping of the specific zones' sequences marked in Figure 3(b) shows that these specific sequences are mainly located in the minor territory of human repeat sequences (Figure 4(b)). Therefore, the specific sequences actually belong to the repeat category. However, it has been separately shown that these specific sequences are different from the ubiquitously distributed human repeat sequences such as Alu and LI (our unpublished data). As another separate analysis, we have found that these sequences are also different from alphoid sequences, which are a major component of human centromeric regions. Core parts of human centromeric regions mainly composed of alphoid sequences have not been included in the standard human genome sequences currently available, because of the difficulty to get contiguous sequences. The minor human territory of interest is colored in black on *U*-matrix (Figure 4(c)) and appears to be split into two parts: a very dark small part and its adjacent gray part.

**Figure 5:**
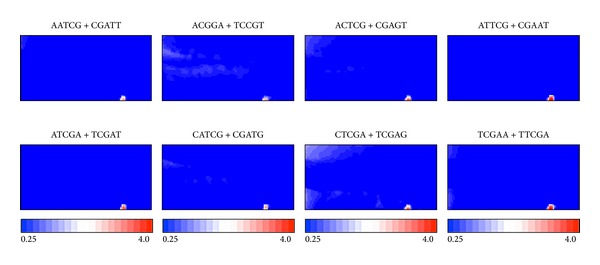
CG-containing pentanucleotides enriched in Sz region. The observed/expected ratio for each pair of complimentary pentanucleotides is calculated as described in Figure 1(c) and indicated in color presented under the panel. The small red region for each pair of complimentary pentanucleotides corresponds to the dark black region in the *U*-matrix listed in Figure 4(c).

In Figure 4(d), we list six examples of pentanucleotides (including a TFB motif) diagnostic for separation between species and/or between repeat and unique sequences. Interestingly, all pentanucleotides show a very high (dark red) or very low (dark blue) occurrence in the minor human repeat territory, again showing its very peculiar oligonucleotide composition. The specific characteristics in this minor repeat territory are further confirmed when we examine the dinucleotide CG-containing pentanucleotides (Figure 5). All these CG-containing pentanucleotides listed are specifically overrepresented in the very dark, small region visualized in *U*-matrix (Figure 4(c)) but evidently underrepresented in all other regions. When we examine the occurrence of all CG-containing pentanucleotides in detail, the CGA-containing pentanucleotides are particularly enriched in the very dark, small region in *U*-matrix, and almost all examples listed in Figure 5 correspond to the CGA-containing pentanucleotides.

The evident underrepresentation of the CG dinucleotide (i.e., CG suppression) is well known in vertebrate genomes and the CG suppression is believed to relate to methylation at CG dinucleotide, which is a well-characterized epigenetic marker. Concerning the CG occurrence level, CpG islands, in which the CG occurrence is clearly higher than in other genomic regions, are well known to have important roles in transcriptional regulation. The sizes of the CpG islands are known to be a few or several hundred bp and, therefore, are clearly different from the size of specific sequences found in the present study (a 100 kb level). Furthermore, CpG islands belong primarily to the unique sequence regions. Therefore, the 100 kb level sequences enriched with the CG-containing pentanucleotides are not the CpG island sequences. As noted above, the CG dinucleotide is a target of methylation and this C methylation is known to have important roles in epigenetic systems. The 100 kb level specific sequences may have important roles that are different from but possibly related to the function of CpG islands. The finding that the CGA-containing pentanucleotides are more preferred in the specific sequences than other CG-containing pentanucleotides may give information for clarifying biological functions of the 100 kb level sequences of interest.

### 4.2. Possible Biological Functions of Sequences with Peculiar Oligonucleotide Composition

As a separate analysis, we have examined the chromosomal locations of sequences belonging to the human specific zones and found a major portion of these 100 kb specific sequences to be derived from pericentromeric heterochromatin regions (data not shown), as supporting the previous finding [17]. Pericentromeric regions form the heterochromatin structure “chromocenter” in interphase nuclei. Chromocenter was once thought to be stable in composition and transcriptionally inert but has recently been shown to be surprisingly dynamic [24–28]. Mouse centromere-derived double-stranded transcripts appear to be involved in establishing the heterochromatin structure [24], and Dicer-related RNA interference machinery is involved in the formation of the centromeric heterochromatin structure in higher vertebrate cells [29]. A strand-specific burst in transcription of mouse pericentromeric satellites is required for chromocenter formation during early mouse development [27], and long nuclear noncoding RNA transcribed from the periphery of pericentromeric heterochromatin has recently been reported [30]. Because the centromere RNA has been shown to be a key component for the assembly of nucleoproteins at the nucleolus and centromere [31, 32], the notable clustering of TFB motifs in the pericentromeric regions should provide novel knowledge about the higher order of nuclear organization.

In Figure 2, specific regions are mainly observed for the human genomic sequences. This appears to be related to the finding that the human specific sequences are mainly derived from the pericentromeric heterochromatin regions. In the case of mice, their chromosomes are acrocentric and the highly repetitive sequences in their pericentromeric regions are less represented in the reported genome sequence than for the human genome. When more sequences of the mouse pericentromeric regions will become available, comparative analyses of their sequences should provide novel information concerning biological significance of 100 kb level sequences with the very peculiar oligonucleotide compositions. In the present study, we have analyzed 100 kb sequences, but the analyses of 50 kb sequences give similar results (data not shown).

### 4.3. Other Applications of BLSOM and Future Prospects

BLSOM can classify genomic sequences according to species with no information other than oligonucleotide frequencies. Because the classification and visualization power is very high, BLSOM is a powerful bioinformatics tool for extracting a wide range of information from a large amount of genomic sequences. A wide variety of oligonucleotide sequences function as genetic signals (e.g., regulatory signals for gene expression). We have found that occurrence levels of oligonucleotide sequences corresponding to important functional signals (e.g., TFB motif sequences) are often biased significantly from the occurrence levels found in a major portion of the human genome and are diagnostic for the specific zones visualized in Figures 2 and 3. When we systematically characterize in advance the known signal sequences of various species with enough experimental data with BLSOMs, we may develop an* in silico* method of signal prediction, which is most useful for genomes that are sequenced but for which little additional experimental data are available. Because the number of such genomes has increased rapidly, development of the* in silico* method has become increasingly important. Functional signals, such as transcription-regulatory signals, are typically longer than pentanucleotides, and therefore analyses of longer oligonucleotides become important. To conduct BLSOM with longer oligonucleotides such hexa- and heptanucleotides (4,096- and 16,384-dimensional data) for a massive amount of genome sequences currently available, a large-scale computation using a high-performance supercomputer will become essential, and the BLSOM algorithm is suitable for a high level of parallel computing.

One important application of BLSOM to genome informatics is the use for metagenome analyses. Most environmental microorganisms cannot be cultured easily under laboratory conditions. Genomes of uncultured organisms have remained mostly uncharacterized and are thought to contain a wide range of novel genes of scientific and industrial interest [33–38]. Metagenomic approaches, which are analyses of mixed populations of uncultured microbes, have been developed to identify novel and industrially useful genes and to study microbial diversity in a wide variety of environments. With the metagenomic approach, genomic DNAs are extracted directly from an environmental sample containing multiple organisms, and the DNA fragments are cloned and sequenced. This is a powerful strategy for comprehensive analysis of biodiversity in an ecosystem. However, for a simple collection of many sequence fragments, the conventional phylogenetic method cannot predict from what phylotypes individual sequences are derived or the phylogenetic novelty of the individual sequences. Traditional methods of phylogenetic assignment have been based on sequence homology searches and therefore inevitably focused on well-characterized genes, for which orthologous sequences required for constructing a reliable phylogenetic tree are available. However, most of the well-characterized genes are not industrially attractive. BLSOM is an alignment-free clustering method, and thus is the most suitable method for this metagenomics analysis.

For phylogenetic classification of species-unknown sequences obtained from environmental and clinical samples, we have constructed BLSOMs in advance with all available sequences from species-known prokaryotes and eukaryotes, as well as from viruses and organelles, and found that the sequences are clustered (self-organized) according to phylotypes with high accuracy [16]. By mapping a large number of environmental metagenomic sequences on the large-scale BLSOM, we can predict phylotypes of these environmental sequences [39]. Because BLSOM does not require orthologous sequence sets, this alignment-free method can provide a systematic strategy for revealing microbial diversity and relative abundance of different phylotype members of uncultured microorganisms including viruses in an environmental sample [39]. Actually, as collaborative studies with experimental research groups, we have used the BLSOM for phylogenetic classification of genomic sequence fragments obtained from mixed genomes of uncultured microbes in environmental samples [18, 40, 41]. We have recently found that the addition of a large number of computer-generating random sequences can classify the metagenomic sequences according to phylotypes [42]. In addition, BLSOM with oligopeptide composition can classify protein sequences mainly according to function [18].

## 5. Conclusions

Because of the remarkable progress of various high-throughput measuring instruments, a massive amount of various data other than sequence data has been accumulated. Complex data can be represented by a high-dimensional multivariate data. BLSOM can analyze a massive amount of high-dimensional multivariate data because the algorithm is suitable for high-level parallel computing. BLSOM can support efficient knowledge discoveries from such big data, showing that the BLSOM is a timely bioinformatics method in the era of big data studies in bioscience. In the present study, we characterized vertebrate genomes using BLSOM. We first analyzed pentanucleotide compositions in 100 kb sequences derived from a wide range of vertebrate genomes and then the compositions in the human and mouse genomes in order to investigate a method for detecting differences between the closely related genomes. BLSOM can recognize the species-specific key combination of oligonucleotide frequencies in each genome, which is called a “genome signature,” and the specific regions specifically enriched by transcription-factor-binding sequences.
